# Genome reannotation of the lizard *Anolis carolinensis* based on 14 adult and embryonic deep transcriptomes

**DOI:** 10.1186/1471-2164-14-49

**Published:** 2013-01-23

**Authors:** Walter L Eckalbar, Elizabeth D Hutchins, Glenn J Markov, April N Allen, Jason J Corneveaux, Kerstin Lindblad-Toh, Federica Di Palma, Jessica Alföldi, Matthew J Huentelman, Kenro Kusumi

**Affiliations:** 1 School of Life Sciences, Arizona State University, PO Box 874501, Tempe, AZ, 85287-4501, USA; 2 Neurogenomics Division, Translational Genomics Research Institute, 445 N. 5th St., Phoenix, AZ, 85004, USA; 3 Broad Institute of MIT and Harvard, Cambridge, MA, 02142, USA; 4 Science for Life Laboratory Uppsala, Department of Medical Biochemistry and Microbiology, Uppsala University, Uppsala, Sweden

**Keywords:** Annotation, Lizard, *Anolis carolinensis*, Transcriptome, Genome, RNA-Seq, Gene, Vertebrate, Embryo, Tissue-specific

## Abstract

**Background:**

The green anole lizard, *Anolis carolinensis*, is a key species for both laboratory and field-based studies of evolutionary genetics, development, neurobiology, physiology, behavior, and ecology. As the first non-avian reptilian genome sequenced, *A. carolinesis* is also a prime reptilian model for comparison with other vertebrate genomes. The public databases of Ensembl and NCBI have provided a first generation gene annotation of the anole genome that relies primarily on sequence conservation with related species. A second generation annotation based on tissue-specific transcriptomes would provide a valuable resource for molecular studies.

**Results:**

Here we provide an annotation of the *A. carolinensis* genome based on *de novo* assembly of deep transcriptomes of 14 adult and embryonic tissues. This revised annotation describes 59,373 transcripts, compared to 16,533 and 18,939 currently for Ensembl and NCBI, and 22,962 predicted protein-coding genes. A key improvement in this revised annotation is coverage of untranslated region (UTR) sequences, with 79% and 59% of transcripts containing 5’ and 3’ UTRs, respectively. Gaps in genome sequence from the current *A. carolinensis* build (Anocar2.0) are highlighted by our identification of 16,542 unmapped transcripts, representing 6,695 orthologues, with less than 70% genomic coverage.

**Conclusions:**

Incorporation of tissue-specific transcriptome sequence into the *A. carolinensis* genome annotation has markedly improved its utility for comparative and functional studies. Increased UTR coverage allows for more accurate predicted protein sequence and regulatory analysis. This revised annotation also provides an atlas of gene expression specific to adult and embryonic tissues.

## Background

Recent advances in sequencing technologies and *de novo* genome assembly algorithms have greatly reduced the time, cost, and difficulty of generating novel genomes [1]. This has led to organized efforts to sequence a representative species from all major vertebrate taxa, referred to as the Genome 10K Project [2], as well as a similar project to sequence five thousand insect genomes, the i5K project [3]. While these efforts have the potential to transform comparative studies, many applications including studies of biological function will be limited without quality genome annotations. Genome annotations of newly sequenced species initially rely primarily on *ab initio* gene predictions and alignment of reference transcripts of related species; however, the quality of gene models is greatly improved when incorporating same species transcriptomic sequencing [4]. In particular, information from high-density next-generation RNA sequencing, i.e., deep transcriptomes, greatly improves even well-annotated genomes [5].

While 39 mammalian genomes and 3 avian genomes have been published [6], whole genome sequences have only recently been available for non-avian reptiles. The first published non-avian reptilian genome was that of a squamate, the lizard *Anolis carolinensis* (Anocar2.0 assembly) [7]. Subsequently, releases of draft genomes from another squamate, the Burmese python, *Python molurus bivittatus*, [8] and three crocodilian species: the American alligator, *Alligator mississippiensis*, the gharial *Gavialis gangeticus*, and the saltwater crocodile *Crocodylus porosus*[9] were published. As an emerging model system with its genome sequence available, the green anole has already proved useful in a variety of fields including comparative genomics [10-13], functional genomics [14,15], behavior [16,17], evolutionary genetics [18,19], and development and evolution [20,21]. In all of these areas of research, the green anole genome, in combination with avian and mammalian data, provides a key perspective on conserved and divergent features among amniotes.

Currently, the public databases of the National Center for Biotechnology Information (NCBI), Ensembl, and University of California, Santa Cruz (UCSC) have devoted anole genome portals. NCBI and Ensembl provide first generation genome annotations, which are based primarily on conservation with other species [7]. These first generation annotations rely heavily on conservation of protein-coding sequences, and as such, predicted green anole genes generally lack untranslated regions (UTRs) and often do not contain start and/or stop codons. Furthermore, alternative splice forms and evolutionarily divergent orthologues are not represented in the first generation annotations. These issues have limited the ability of researchers to carry out comparative and functional genomic studies based on the *A. carolinensis* genome sequence.

In order to help resolve many of these issues, here we present a second generation revised annotation based on a foundation of 14 *de novo* deep transcriptomes and published cDNA sequences. We used a customized pipeline based on the Program to Assemble Spliced Alignments (PASA) [5,22-24], EVidenceModeler (EVM) [4] and MAKER2 [25,26] to combine the following data: i) *de novo* and reference based assemblies of 14 RNA-Seq transcriptomes, ii) 7 publicly available EST libraries, iii) RefSeq alignments of the available vertebrate transcripts, iv) RefSeq alignments of zebrafish, *Xenopus* frog, chicken, mouse, and human protein sequences, v) NCBI and Ensembl current annotations, and vi) *ab initio* gene predictions based on analysis by SNAP and Augustus [27-29].

## Results and discussion

### De novo transcriptome generation and assembly

We carried out RNA-Seq to generate 11 adult tissue and 3 embryonic transcriptomes (Table 1). Strand-specific directional sequences were generated from adrenal gland, brain, dewlap skin, heart, liver, lung, ovary, and skeletal muscle. RNA-Seq generated by directional library construction can be used to distinguish between coding transcripts and antisense noncoding transcripts. The adrenal, lung, liver and skeletal muscle samples were derived from a single male individual (Additional file 1: Table S1). The brain, dewlap skin, heart, and ovary samples were pooled from several individuals (Additional file 1: Table S1). Standard non-directional RNA-Seq libraries were prepared from regenerating tail and embryonic tissues. Lizards including the green anole can regenerate their tail following autotomy, or self-amputation [30]. Regenerating tissues from 3 tails at 15 days post-autotomy were divided into pools of the regenerating epithelial tip and the adjacent tail base. RNA-Seq was also performed on the original autotomized tail from those same animals. Embryos between zero to one day after egg laying (28 and 38 somite-pair stages) were analyzed individually by standard RNA-Seq as well as pooled for directional library construction and sequencing. More than 762 million paired-end reads were generated from these adult and embryonic tissue samples (Table 1).

**Table 1:** **Overview of *de novo *transcript assembly for *A. carolinensis *based on RNA-Seq data from 14 adult and embryonic tissues and deposited EST sequence data**

***De novo *RNA-Seq**	**# Reads**	***De novo *assembled transcripts**	**Transcripts aligning to Anocar2.0 assembly**	**PASA assembled transcripts**
Embryo-28 somite stage	52,548,024	83,627	81,032	22,670
Embryo-38 somite stage	55,048,179	99,578	95,753	24,595
Regenerating tail tip	122,099,352	92,275	88,150	22,278
Regenerating tail base	31,721,054	78,005	73,516	24,897
Original tail	109,404,060	96,450	91,601	20,240
Adrenal	55,858,836	110,349	101,449	20,482
Brain	32,518,977	203,519	192,407	33,912
Dewlap skin	31,785,178	81,598	76,866	25,853
Embryos (pooled)	59,681,427	118,949	110,124	19,969
Heart	34,068,834	154,255	144,617	26,582
Liver	50,782,350	89,010	81,441	21,549
Lung	48,723,049	272,071	255,035	37,985
Ovary	35,139,647	80,306	75,807	26,827
Skeletal Muscle	42,707,477	75,006	69,250	18,857
**Total**	**762,086,444**	**1,634,998**	**1,537,048**	**346,696**
**EST Library (NCBI)**	**# Sequences**	**NCBI defined UniGene groups**	**Transcripts aligning to Anocar2.0 assembly**	**PASA assembled transcripts**
Brain	19,139	5,631	9,991	1,715
Dewlap skin	19,809	5,453	10,180	2,216
Embryo	38,923	8,714	9,991	4,158
Mixed Organ	19,863	5,657	9,327	2,053
Ovary	19,410	5,467	7,394	3,737
Regenerating Tail	19,851	6,751	11,064	6,757
Testis	19,807	4,261	8,677	2,594
**Total**	**156,802**	**41,934**	**66,624**	**23,230**

**Figure 1:**
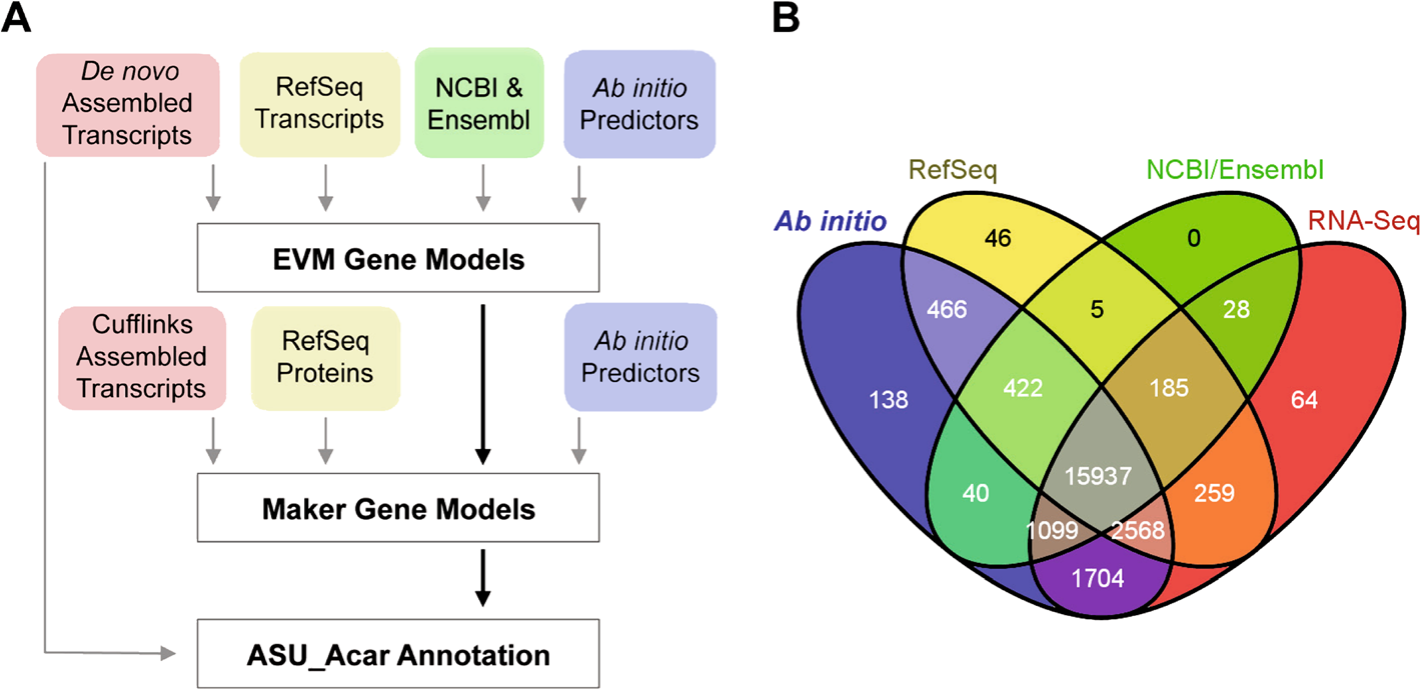
**A. Diagram of the bioinformatic pipeline for the *A. carolinensis *reannotation. B.** Venn diagram illustrating the sources of data for the *A. carolinensis* reannotation. *Ab initio*, algorithm based gene predictions using Augustus and SNAP [26-28]. RefSeq, alignments of zebrafish, *Xenopus* frog, chicken, mouse, and human protein and available vertebrate transcripts to the Anocar2.0 genome assembly. NCBI/Ensembl, combined data of *A. carolinensis* genome annotations from NCBI ref_Anocar2.0 and Ensembl Build 65. RNA-Seq, transcriptomic data from analysis of 14 adult and embryonic tissues.

The pipeline for *de novo* assembly of RNA-Seq data involved two steps. First, strand-specific transcriptome sequence libraries were assembled using Trinity (Figure 1A) [31]. Standard non-directional RNA-Seq libraries were assembled using ABySS and Trans-ABySS [32-34]. In total, this generated more than 1.62 million *de novo* assembled transcript contigs. Second, these assembled contigs were aligned to the *A. carolinensis* Anocar2.0 assembly [7] using the gmap tool within PASA, with the aim of i) eliminating sequences not aligning to the genome and ii) merging *de novo* assembled sequences to remove redundancy. We observed that over 94% of these sequences aligned to the green anole genome at a cutoff of 95% identity and 90% transcript coverage. This first step of the *de novo* assembly pipeline reduced the number of RNA-Seq based transcript contigs down to 669,584.

As part of the *A. carolinensis* genome sequencing effort, EST sequences were generated from five adult organs (brain, dewlap skin, ovary, regenerated tail, and testis), embryo, and a seventh mixed organ library that included heart, kidney, liver, lung, and tongue [7]. These EST sequences were introduced at the second step of this pipeline and aligned to the *A. carolinensis* Anocar2.0 assembly using gmap, identifying another 35,188 transcript contigs not present from the RNA-Seq deep transcriptomic data. This yielded a total of 704,772 transcript contigs that were then used as the basis of the second generation *A. carolinensis* genome annotation (Table 1).

### Generating a revised annotation of the A. carolinensis genome

The reannotation of the *A. carolinensis* genome incorporates four classes of evidence that were combined using the EVM tool (Figure 1A) [4]. First, the 704,772 *de novo* assembled transcript contigs were given the highest weight to generate the revised annotation. Second, two *ab initio* gene prediction tools, SNAP and Augustus [27-29], were trained using a subset of the PASA transcriptome assemblies after removing redundancy using CD-HIT [35]. In brief, 9,064 *A. carolinensis* coding sequences were used to train SNAP, and 1,041 complete predicted protein sequences were used to train Augustus. Third, the first generation *A. carolinensis* gene annotations from NCBI ref_Anocar2.0 (abbreviated as NCBI) and Ensembl Build 65 (abbreviated as Ensembl) were used as an input to EVM. Finally, regions of alignment to RefSeq homologous transcript sequences from the UCSC Genome Bioinformatics portal were also incorporated into the EVM predictions.

Since EVM currently generates only a single protein-coding sequence for each gene and the transcript evidence requires at least 90% alignment to the genome, further steps were necessary to improve the annotation. First, the RNA-Seq reads were aligned to the Anocar2.0 assembly using TopHat and reference guided assemblies were completed using Cufflinks. Second, the EVM predictions, the Cufflinks assemblies, as well as zebrafish, *Xenopus* frog, chicken, mouse, and human protein alignments, were used as input into MAKER2 to annotate novel genes, extend UTR sequence, and annotate alternative splicing (Figure 1A). These models were updated to further incorporate UTR sequences and alternate splice forms present in the *de novo* assembled transcripts described above. We have named this second generation annotation for *A. carolinensis* ASU_Acar version 2.1 (abbreviated as ASU).

### Sources for genome reannotation

The improvements in the ASU annotation derive from multiple sources. The largest group of annotated genes, 69% or 15,937, were based on all sources of data, and an additional 30% (6,776) were based on two or three sources of data (Figure 1B). In addition, RNA-Seq was a key source of data for this reannotation, contributing to 95% of all gene predictions. Only 1% of predicted genes were based solely on one source of data (transcriptome, NCBI or Ensembl annotation, RefSeq alignment, or *ab initio* predictions). The *ab initio* gene predictions, which do not make use of any empirically derived data, contribute less than 1% (138) of the gene predictions for this reannotation. Since both the first and second generation annotation pipelines rely on open reading frame and coding sequence predictions, noncoding transcripts are likely underrepresented. The generation of long noncoding and microRNA-Seq data and sampling of more tissues by the research community will contribute to improved *A. carolinensis* genome annotations in the future.

### Improvements in gene annotation

**Figure 2:**
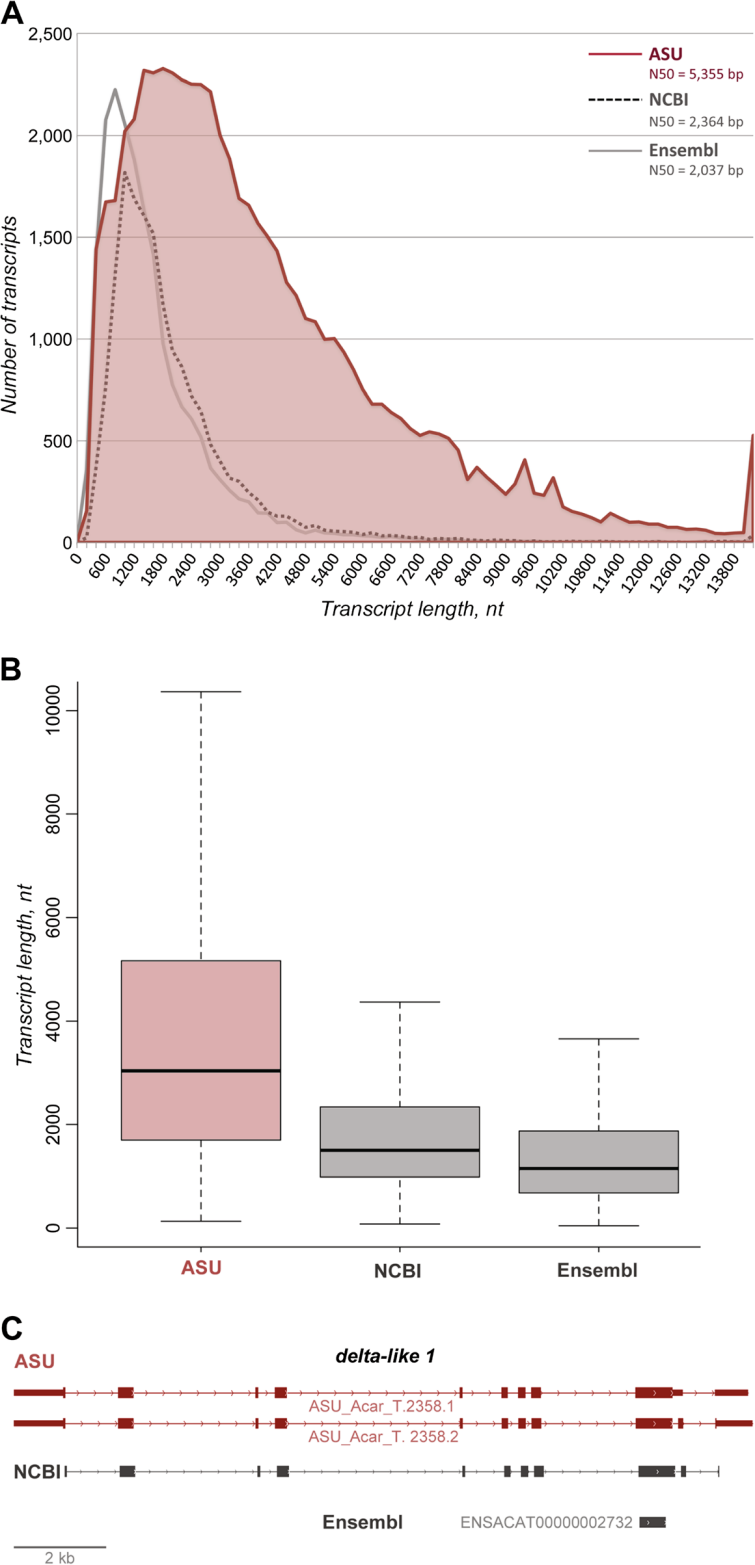
**Increased N50 transcript length and number of predicted transcripts in the ASU annotation.A. **The distribution of transcript lengths is shown for the ASU, NCBI and Ensembl genome annotations. The ASU annotation transcript N50 length of 5,355 bp is greater than values for the first generation annotations from Ensembl (2,037 bp) and NCBI (2,364 bp). **B**. A boxal plot showing the median (horizontal line) and boundaries for the 25^th^ and 75^th^ percentiles (box) as well as the range for the ASU, NCBI, and Ensembl predicted transcripts. **C**. The Notch ligand *dll1* is an example of gene whose annotation has been markedly improved in the ASU annotation.

To quantify the differences between the first and second generation genome annotations, we compared ASU with the NCBI and Ensembl annotations. First, the ASU annotation has identified more genes than either NCBI or Ensembl (22,962 vs. 15,645 in NCBI and 17,792 in Ensembl; Table 2). Second, the ASU annotation greatly increases the number of annotated transcript isoforms (59,373 vs. 16,533 for NCBI and 18,939 for Ensembl; Table 2). Third, predicted transcripts in the ASU annotation appear to be more complete in a number of different parameters. Of the 59,373 annotated transcript isoforms, 90% (53,401) are predicted to be complete protein-coding sequences. Furthermore, 59% (34,926) are predicted to contain 3’ UTR sequences, and 79% (46,782) to contain 5’ UTR sequences (Table 2). In addition, the ASU annotation greatly improves transcript lengths (5,355 bp vs. 2,364 bp for NCBI and 2,037 bp for Ensembl; Figure 2A & B; Table 2). An example of the improvements in gene annotation is evident the Notch pathway ligand, *delta-like 1* (Figure 2C; gene symbol *dll1* following guidelines of the *Anolis* Gene Nomenclature Committee) [36].

**Table 2:** **Comparison of ASU, NCBI and Ensembl gene annotations of the *A. carolinensis *genome**

**Overview**	**ASU**	**NCBI**	**Ensembl**
Annotated genes	22,962	15,645	17,792
Annotated transcript isoforms	59,373	16,533	18,939
Annotated isoforms/gene	2.59	1.06	1.06
**Annotated Transcripts**			
All transcript isoforms	59,373	16,533	18,939
Transcripts with start & stop codons	53,401	14,667	4,170
Transcripts missing start or stop codon	5,972	1,866	14,769
Single exon transcripts	2,070	983	364
Transcript N50 length	5,355	2,364	2,037
Average coding sequence length	1,964	1,701	1,531
**Exons**			
Total number of exons	229,204	156,742	174,545
Exons with start with codon	29,677	13,512	5,971
Exons without start or stop codon	168,367	128,486	158,935
Exons with stop codon	29,727	13,779	9,278
Exons/annotated transcript	12.05	10.11	9.62
Average exon length	170	170	160
Total exon length	38,902,806	26,658,387	27,910,718
**3' UTR**			
Total transcripts with 3'UTR	34,926	5,861	0
Average length of transcripts with 3'UTR	1,168	456	0
Total 3'UTR sequence length	40,798,794	2,674,388	0
**5' UTR**			
Total transcripts with 5'UTR	46,782	6,168	0
Average length of transcripts with 5'UTR	244	86	0
Total 5'UTR sequence length	11,422,626	527,454	0
**Introns**			
Total number of introns	192,418	141,362	155,949
Average intron length	4,525	4,463	2,553
Total intron sequence length	870,771,088	630,937,171	398,124,572

### Assignment of gene orthology

Identification of orthologous relationships between genes in *A. carolinensis* and other vertebrate model systems is a key step in comparative studies. However, this is a complex task due to gene deletions and genome duplications and rearrangements during vertebrate evolution. For protein-coding genes, metrics have been proposed [36] that consider both protein sequence similarity and synteny conservation. For comparison of ASU annotation, we have used the current orthology assignments in the NCBI and Ensembl gene models. Given the longer transcript lengths in the ASU annotation we identified that 16,303 genes overlapped with Ensembl predicted genes and 16,908 overlapped with NCBI predicted genes (Additional file 2: Table S2).

However, this comparison left 5,246 ASU predicted genes with no orthology assignment based on NCBI or Ensembl annotations. Gene orthology for these remaining predicted genes were next evaluated by Blast2GO against the vertebrate RefSeq database [37-39]. This analysis demonstrated that 56% of these predicted genes (2,928/5,246) had a Blast2GO Expect (E) value score of at least 10^-3^ with a vertebrate gene, which is suggestive of a potential orthologue (Table 3; Additional file 3: Table S3). Of these predicted genes, 90% (2,627/2,928) contain multiple exons with an average of 6.4 exons/gene and a N50 value of 2,157 bp. These may reflect genes that have been newly identified in the ASU annotation but were missing in the NCBI and Ensembl annotations. The remaining 10% of the predicted genes (301/2,928) contain only a single annotated exon, which could result from gaps in the Anocar2.0 reference genome assembly. The remaining group of genes (2,318/5,246) aligned to the Anocar2.0 assembly but had poor vertebrate homology. This group may include novel lizard genes and rapidly diverging genes such as noncoding RNAs.

**Table 3:** ***A. carolinensis *genes that are unique to the ASU annotation and have vertebrate orthologues**

**Gene**	**ASU**
Annotated genes	2,928
Annotated transcript isoforms	3,612
Annotated isoforms/gene	1.23
**Transcript**	
All transcript isoforms	3,612
Transcripts with start & stop codons	2,698
Transcripts missing start or stop codon	914
Single exon transcripts	301
Transcript N50 length	2,157
Average coding sequence length	1,182
**Exon**	
Total number of exons	18,921
Exons with start with codon	2,468
Exons without start or stop codon	13,901
Exons with stop codon	2,300
Exons/annotated transcript	6.35
Average exon length	188
Total exon length	3,569,265
**3' UTR**	
Total transcripts with 3'UTR	1,323
Average length of transcripts with 3'UTR	761.2
Total 3'UTR sequence length	1,007,040
**5' UTR**	
Total transcripts with 5'UTR	1,816
Average length of transcripts with 5'UTR	238.7
Total 5'UTR sequence length	433,533
**Intron**	
Total number of introns	15,835
Average intron length	5,304
Total intron sequence length	83,999,254

### Transcripts with vertebrate homology not present in the Anocar2.0 genome assembly

Given a 7.1x genome coverage for the *A. carolinensis* Anocar2.0 assembly, only 81% of the 2.2 Gbp genome is predicted to be included in the current contig sequences [7]. In addition, approximately 30% of the *A. carolinensis* genome consists of repetitive mobile element sequences, which leads to a lower than typical N50 given the sequencing depth. Thus, some transcripts identified by RNA-Seq analysis would not align to the Anocar2.0 assembly, and these transcripts would not included in the ASU annotation. This category of genes missing from the Anocar2.0 assembly may include important developmental or regulatory genes.

We developed a pipeline to analyze the genes poorly represented in the Anocar2.0 assembly (see Materials and Methods). Starting with 638,802 *de novo* assembled contigs, the pipeline reduced this group down to 29,706 and increased the N50 value from 349 bp up to 2,074 bp. Next, these 29,706 contigs were analyzed by Blast2GO to identify homology to vertebrate RefSeq entries with an E-value cutoff of 10^-3^ (Additional file 3: Table S3). The majority of these contigs (56% or 16,542/29,706) could be matched to 6,695 distinct vertebrate orthologues (Additional file 4: Table S4; Additional file 5: Figure S1).

Analyzing these matching contigs further, we were able to identify matches with 30% of the contigs (4,910/16,542) against the 2,233 *A. carolinensis* RefSeq proteins. This suggests that these transcript contigs that matched *A. carolinensis* RefSeq proteins but failed to align to the Anocar2.0 assembly contain genes that are partially represented on the genome or are interrupted by large gaps in the scaffolds. The remaining 70% (11,632/16,542) of these contigs mapped with highest scores to other vertebrate species (Additional file 5: Figure S1). This is likely due to the incomplete state of the *A. carolinensis* RefSeq libraries. Genes missing from *A. carolinensis* annotations can be attributed to gaps in the Anocar2.0 assembly; misassembly in genome scaffolds would interrupt contiguous alignments of transcripts at contig sequence boundaries. Given these observations, additional sequencing to increase coverage of the *A. carolinensis* genome would improve future annotations.

### High quality genome annotation requires both whole genome and transcriptome sequencing

Next-gen sequencing technologies are accelerating the rate at which whole genome assemblies are being completed. Among the non-avian reptiles, genomes drafts have been reported for the snake *P. m. bivittatus*, [8], and the crocodilian reptiles *A. mississippiensis*, *G. gangeticus*, and *C. porosus*[9]. However, the reannotation of the *A. carolinensis* genome has highlighted the relevance of collecting deep transcriptome data from a diverse array of tissues. For evolutionary genetic studies, maximizing the coverage of protein coding sequences is essential, and prediction of these regions based on whole genome sequences alone is challenging. Furthermore, identification of cis-regulatory regions is aided by improved gene annotations, since the 5’ untranslated sequences near the promoter are poorly conserved compared to protein coding sequences. Alternate splicing is a mechanism that greatly increases the diversity of transcripts from vertebrate genomes, but identification of isoforms requires transcript sequence data from a variety of tissues. Reannotation of the anole genome suggests that for the Genome 10K Project [2], it will be necessary to carry out both whole genome and transcriptome sequencing efforts in order to achieve the comparative genomic goals.

## Conclusions

With the release of the *A. carolinensis* genome, along with a first generation annotation provided by NCBI and Ensembl, a growing foundation of genomic resources are available for the anole reptilian model. Furthermore, genome annotations of this key reptilian model provide a valuable resource for genomic comparison with mammals, such as mice and humans. Using RNA-Seq, we have improved the genome annotation for *A. carolinensis*, which includes 59,373 transcript isoforms, many of which are complete with UTR sequences. *De novo* transcriptome assembly also identified 16,542 transcripts that are not well represented on the current Anocar2.0 genome build. This revised genome annotation and available transcriptomic sequences provide a resource for vertebrate comparative and functional studies. This work also highlights the need for additional genomic sequencing of *A. carolinensis* to fill in gaps and extend scaffolds, as well as further transcriptomic sequencing of additional tissues.

## Materials and methods

### Animals

All animals were maintained and research carried out according to Institutional Animal Care and Use Committee guidelines of Arizona State University. *Anolis carolinensis* lizards were purchased from approved vendors (Charles D. Sullivan Co., Inc., Nashville, TN; Marcus Cantos Reptiles, Fort Myers, FL) and were housed at 70% humidity. Lighting and temperature were maintained for 14 hours at 28°C daylight and 10 hours at 22°C night. Adult tissues were collected immediately after euthanasia. Eggs were collected within one day of laying, typically at the 25-30 somite pair stage.

### RNA-Seq

Samples for RNA-Seq, including embryos, regenerating tail, original tail, dewlap skin, brain, heart, lung, liver, adrenal, ovary, and skeletal muscle were collected for extraction using the total RNA protocol of the miRVana kit (Ambion). For the regenerating tail, original tail, 28 and 38 somite-pair staged embryos, total RNA samples were prepared using the Ovation RNA-Seq kit (NuGEN) to generate double stranded cDNA. Illumina manufacturer protocols were followed to generate paired-end sequencing libraries. Sequencing was carried out on the Illumina HiSeq 2000 using paired-end chemistry with read lengths of 104 base pairs. Strand-specific RNA sequencing libraries were prepared for adrenal, brain, dewlap, pooled 28 and 38 somite staged embryos, heart, liver, lung, skeletal muscle, and ovary RNA samples using the dUTP protocol [40]. The dUTP strand-specific libraries were sequenced on the Illumina HiSeq using paired-end chemistry with read lengths of either 76 or 101 bp.

### De novo assembly

Non-directional RNA-Seq data was assembled using the ABySS and Trans-ABySS pipeline [31-33]. Each sample was assembled in ABySS using every 5^th^ kmer ranging from 26bp to 96bp. These assemblies were then combined using trans-ABySS to create a merged assembly with reduced redundancy. This merged assembly was then mapped to the genome using BLAT inside trans-ABySS. *De novo* assembled contigs were then filtered to require at least 90% coverage of the contig to the genome and at least one 25 bp gap. Because of its ability to utilize stranded information, Trinity was used to assemble the strand-specific RNA sequencing data using default parameters [30].

### PASA alignment assembly

The *de novo* assembled transcripts from ABySS/Trans-ABySS and Trinity [31], as well as the contigs from the EST data sets, were then assembled using the PASA reference genome guided assembly. Seqclean was first used to remove Illumina adapters and any contaminants listed in the UniVec databases from the *de novo* assembled transcripts and the EST libraries. PASA alignment and assembly was then executed using default parameters and utilizing the strand-specific data when possible [5,22-24].

### Ab initio training (PASA/CD-HIT) and prediction

In order to train *ab initio* gene prediction algorithms, a set of high confidence transcripts were extracted from the PASA assemblies from each RNA sequencing data set. These transcripts were then combined and redundancy removed using CD-HIT [35,40]. This set of transcripts was then used to train gene identification parameters for Augustus [28,29], as well as SNAP [27] inside MAKER2 [25,26]. Each gene finder was then run to produce a set of *ab initio* predictions for the *A. carolinensis* genome.

### EVM annotation combiner/PASA updates

EVidenceModeler [4] was utilized to combine *ab initio* gene predictions from Augustus and SNAP, the Ensembl Build 65, and the NCBI ref_Anocar2.0 gene predictions, in combination with UCSC reference protein alignments and *A. carolinensis* transcriptomic data from the PASA assembles. This initial annotation ignored alternate splicing and UTRs. Cufflinks assemblies derived from TopHat alignments of the raw reads, as well as human and chicken RefSeq protein alignments carried out using Exonerate, were used to guide a MAKER2 annotation update to include novel genes, UTR sequences and alternative splicing isoforms [41-48]. PASA was then again utilized to update this initial genome annotation from EVM and MAKER2 to add alternate splicing and UTR sequences based on transcript data [4,5,22-24,26]. Orthologues were then assigned to these annotations through finding overlapping gene annotations from NCBI or Ensembl gene models. ASU gene predictions that did not have an overlap with NCBI or Ensembl genes were then assigned orthology by identifying the most similar vertebrate RefSeq protein using blastx inside Blast2GO [37-39].

### Reference guided assembly

To improve the annotations of genes located in regions interrupted by gaps in the genomic assembly, sequencing reads were used for reference guided transcript assembly. Reads from each sample were first trimmed based on quality and mapped to the *A. carolinensis* genome using Bowtie and TopHat as described previously [42,44,45]. Read alignments and the EVM gene models were then used to guide a Reference Annotation Based Transcript (RABT) assembly using Cufflinks version 2.0.0 [43,46,48].

### Analysis of transcript contigs that failed to align to the Anocar2.0 genome assembly

All tissue-specific contigs that failed to align to the Anocar2.0 genome assembly were processed and assembled using CAP3 [49]. This second assembly step reduces redundancy between the assemblies from each sample and extends any partial transcripts contained within individual sample assemblies. Transcripts were filtered to remove >33% percentage of repetitive sequences using RepeatMasker [50] and remaining transcripts that contained open reading frames longer than 100 amino acids were then extracted from the CAP3 assembly for further analysis. Because CAP3 tended to reconstruct more complete transcripts, these transcripts containing ORFs longer than 100 amino acids were then realigned to the genome using BLAT and alignments covering greater than 70% of the transcript at 90% identity were removed [51]. The filtered transcript contigs were then assigned orthology based on a best blastx match to vertebrate RefSeq proteins using Blast2GO [37-39].

### Annotation files and accession numbers

The ASU_Acar version 2.1 annotation files have been deposited to NCBI and Ensembl for release through their anole-specific portals. Assemblies and the meta-assembly of unmapped transcripts have also been distributed to the *A. carolinensis* community research portals, *AnolisGenome* (http://www.anolisgenome.org) and *lizardbase* (http://www.lizardbase.org).

Accession numbers for non strand-specific RNA-Seq and transcript assemblies: embryo-28 somite [NCBI-GEO: GSM848765; SRA: SRX111311, TSA: SUB139328], embryo-38 somite [NCBI-GEO: GMS848766, SRA: SRX111310, TSA: SUB139329], regenerating epithelial tail tip [SRA: SRX158076, TSA: SUB139331], regenerating tail base [SRA: SRX158077, TSA: SUB139332], tail [SRA: SRX158074, TSA: SUB139330]. Accession numbers for strand-specific RNA-Seq and transcript assemblies: adrenal gland [SRA: SRX145078, SRX146889, TSA: SUB139081], brain [SRA: SRX111454, TSA: SUB139082], dewlap skin [SRA: SRX111451, TSA: SUB139084], embryo pooled [SRA: SRX115247, SRX146888, TSA: SUB139085], heart [SRA: SRX111452, TSA: SUB139086], liver [SRA: SRX112551, TSA: SUB139087], lung [SRA: SRX112552, TSA: SUB139088], ovary [SRA: SRX111453, TSA: SUB139091], skeletal muscle [SRA: SRX112550, TSA: SUB139090], the reassembly of unmapped transcripts from all RNA-seq data [TSA: SUB139333]. Library accession numbers for EST sequence libraries reported previously [7] used for analysis: brain [UniGene: Lib.23338], dewlap skin [UniGene: Lib.23339], embryo [UniGene: Lib.23340], mixed organ [UniGene: Lib.23341], ovary [UniGene: Lib.23342], regenerating tail [UniGene: Lib.23343], testis [UniGene: Lib.23344].

## Competing interests

The authors declare that they have no competing interests.

## Authors' contributions

WE, EH and KK planned the experiments. WE, EH, GM, and KK managed the lizard colony, collected tissue samples and extracted RNA. AA, JC, EH, and MH carried out the nondirectional RNA-Seq. FdP, KL-T and JA supervised the directional RNA-Seq efforts. WE, EH, and KK wrote the manuscript. All authors read and approved the final manuscript.

## Supplementary Material

Additional file 1 **Table S1.** Description of adult and embryonic tissues used for RNA-Seq. Click here for file

Additional file 2 **Table S2.** Orthology assignment of ASU_Acar genes. Click here for file

Additional file 3 **Table S3.** Orthology assignment of unmatched ASU_Acar annotated genes by Blast2GO. Click here for file

Additional file 4 **Table S4.** Orthology assignment of unmapped transcripts by Blast2GO. Click here for file

Additional file 5 **Figure S1.** Blast2GO matches for transcripts poorly aligning to the Anocar2.0 genome assembly. The species with the highest Blast2GO matches are shown. Click here for file
