# Supplementary figures and images for: Genome reannotation of the lizard Anolis carolinensis based on 14 adult and embryonic deep transcriptomes
Source: BMC Genomics. 2013 Jan 23;14:49. doi: 10.1186/1471-2164-14-49 (PMC3561122; doi:10.1186/1471-2164-14-49)

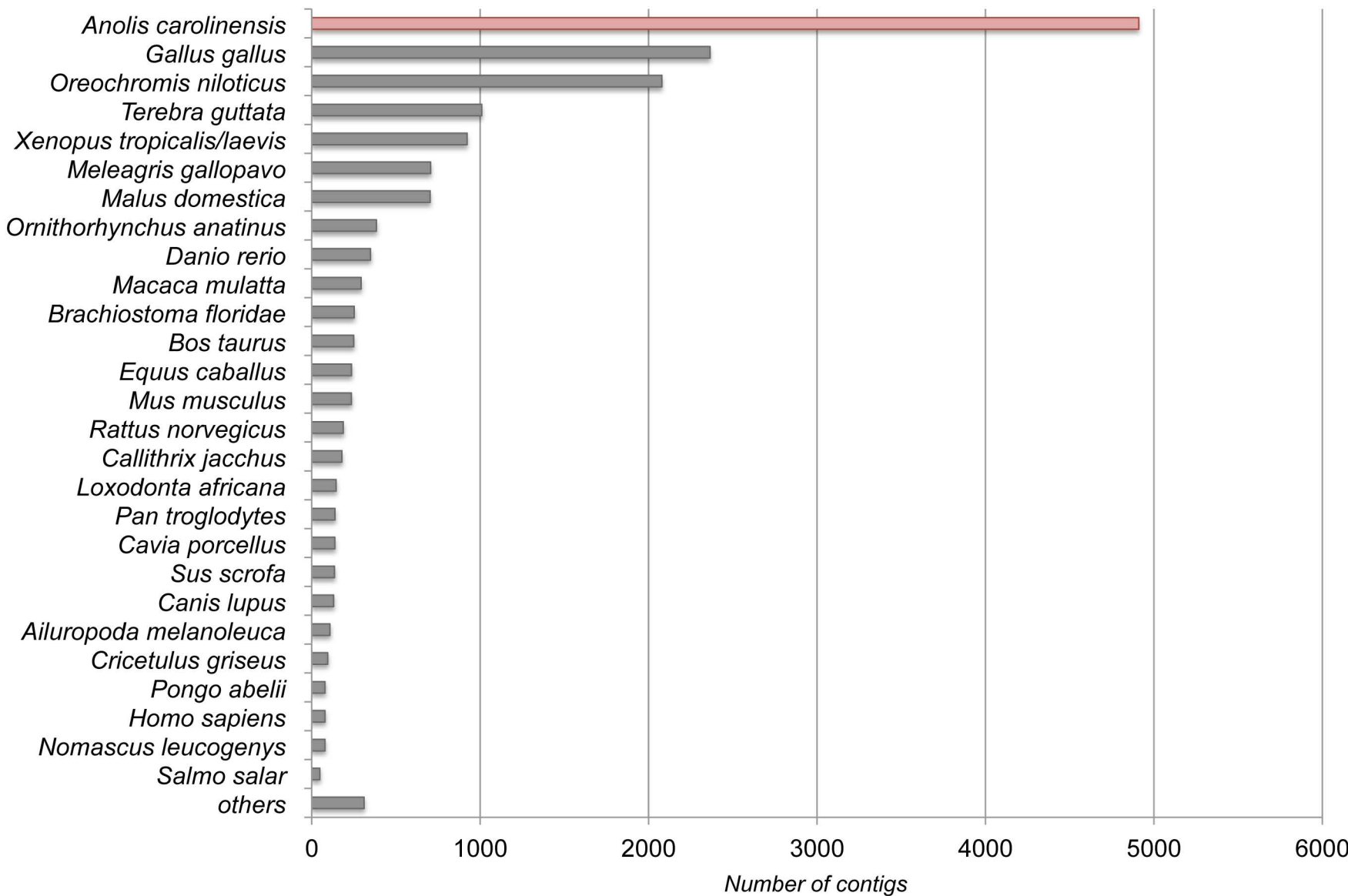

Supplement: Additional file 5 — Figure S1. Blast2GO matches for transcripts poorly aligning to the Anocar2.0 genome assembly. The species with the highest Blast2GO matches are shown. [file 1471-2164-14-49-S5.pdf]
